# The trend of top five types of poisonings in hospitalized patients based on ICD‐10 in the northeast of Iran during 2012–2018: A cross‐sectional study

**DOI:** 10.1002/hsr2.587

**Published:** 2022-04-13

**Authors:** Alireza Banaye Yazdipour, Mohammad Moshiri, Bita Dadpour, Masoumeh Sarbaz, Hamid Heydarian Miri, Saeedeh Hajebi Khaniki, Khalil Kimiafar

**Affiliations:** ^1^ Department of Health Information Management, School of Allied Medical Sciences Tehran University of Medical Sciences Tehran Iran; ^2^ Students' Scientific Research Center (SSRC) Tehran University of Medical Sciences Tehran Iran; ^3^ Department of Medical Records and Health Information Technology, School of Paramedical Sciences Mashhad University of Medical Sciences Mashhad Iran; ^4^ Medical Toxicology Research Center, School of Medicine Mashhad University of Medical Sciences Mashhad Iran; ^5^ Department of Pharmacodynamy and Toxicology, School of Pharmacy Mashhad University of Medical Sciences Mashhad Iran; ^6^ Department of Epidemiology, School of Health Mashhad University of Medical Sciences Mashhad Iran; ^7^ Student Research Committee, Department of Biostatistics, Faculty of Health Mashhad University of Medical Sciences Mashhad Iran

**Keywords:** drug poisoning, ICD‐10, Iran, trends

## Abstract

**Background and Aims:**

Poisoning remains a major health issue in developing countries with high morbidity and mortality rates; also it is one of the most common causes of admission to hospitals. This study aimed to investigate the trend of the top five types of poisonings in hospitalized patients according to the International Statistical Classification of Diseases and Related Health Problems 10th Revision (ICD‐10) in Imam Reza hospital, Mashhad, Iran.

**Methods:**

This was a cross‐sectional study performed from March 20, 2012, until September 22, 2018. We collected data from all patients hospitalized for poisoning admitted to the poisoning center at Imam Reza hospital in northeast Iran. ICD‐10 was adopted to categorize all types of poisonings (T36‐T65). The results obtained were analyzed by SPSS 16.

**Results:**

Thirty‐four thousand eight hundred and ten cases were included. The mean age of the patients was 29.64 ± 14.69 years, of them, 50.7% were males. Benzodiazepine poisoning (T42.4) has the highest frequency among other subcategories and it was more common among females (60.5%). Opium poisoning (T40.0) has the highest mortality rate (5.4%) among other subcategories that is more common in males (72.0%). The mortality associated with narcotics was the highest frequency (2.7%). Suicide (83.6%) was the most common cause of poisoning. Most poisonings occurred in summer (27.4%).

**Conclusion:**

These findings could help health care managers and policymakers develop prevention and educational programs to reduce these poisonings and limit people's easy access to drugs and substances.

## 1 INTRODUCTION

Poisoning is defined as the harm a person is subject to when exposed to drugs, chemicals, or toxins. It is also considered a threat to public health that can lead to morbidity and mortality worldwide.^1^, ^2^ Poisoning is considered to be the second leading cause of death‐related injury in the United States.^3^ Every year, many people around the world are admitted to emergency rooms due to various types of poisoning.^4^ Due to changes in people's lifestyles and their social behavior, the trend of poisoning is changing day by day in today's societies.^5^ Drug poisoning is more common than other poisonings and it can be used intentionally (suicide) or unintentionally (accidental).^6^ Drug abuse is a serious problem in many countries, especially among young men.^7^ Behavioral factors such as cigarette and cannabis use and psychological and personality factors including depression and impulsive and anxious traits, increase the risk of drug abuse day by day, which can lead to drug poisoning.^8^, ^9^

In developing countries, the prevalence of acute intentional and unintentional poisoning varies from 0.2 to 9.3 cases per 1000 individuals annually.^10^ In addition, in recent decades, the rate of morbidity and mortality due to poisoning has almost tripled and about 90% of this morbidity and mortality is due to drugs.^11^ According to a World Health Organization report, approximately 193,460 people died from unintentional poisoning, with 84% of these deaths occurring in low‐ and middle‐income countries.^12^ The pattern of poisoning differs among countries and regions, and this difference depends on various factors such as geography, accessibility, and availability of poison, social, economic, cultural, religious, and legal conditions.^13^, ^14^, ^15^, ^16^

In Iran, poisoning is one of the most common causes of hospitalization and the second leading cause of mortality.^17^ In this developing country, drugs and chemicals are almost easily available to people^18^ and poisoning with drugs and chemicals is common.^4^, ^15^ Natural toxins such as poisonous plants and animals are present in most parts of the country, and drug abuse/addiction is also common.^19^

In Iran, most poisonings occur intentionally and among individuals aged 21–30 years.^4^, ^20^, ^21^ From 1990 to 2015, it is estimated that 40,586 poison‐related deaths occurred throughout Iran.^15^ In addition, in this developing country, the highest frequency of death due to drug poisoning occurs in men.^4^ According to the study on the prevalence of poisoning in Northeastern Iran (Khorasan Razavi, Mashhad) from 2004 to 2013, it was determined the rate of poisoning was increased.^21^ The use of diagnostic classification standards can help healthcare providers, policymakers, researchers, and patients to navigate, understand, and compare healthcare systems and services.^22^, ^23^ One of the used standards in the diagnostic classification is the International Statistical Classification of Diseases and Related Health Problems, the tenth revision (ICD‐10), which has been made to classify diagnoses and other health‐related data of patients hospitalized as alphanumeric codes. Using data coded according to the ICD‐10 facilitates obtaining morbidity information in a standard format and enhances the collection, storage, and analysis of comparable data among countries. Awareness of the types and causes of poisonings based on ICD‐10 can be effective in preventing them. Therefore, the purpose of this study was to investigate the trend of the first five causes of poisonings in northeastern Iran from 2012 to 2018.

## 2 MATERIALS AND METHODS

This cross‐sectional study was performed in the poisoning center of Imam Reza hospital, Mashhad, Iran, from March 20, 2012, to September 22, 2018 (from 1st of Farvardin 1391 to 31st Shahrivar 1397 on the Hijri‐Shamsi date).

Mashhad is the capital of Khorasan Razavi province, which is the largest province in northeast Iran. This province occupies about 118,854 square kilometers of Iran. Imam Reza hospital was the only referral poisoning center affiliated with MUMS (Mashhad University of Medical Sciences) in Mashhad (the northeast of Iran).

We collected retrospective data from all patients hospitalized for poisoning admitted to the poisoning center at Imam Reza hospital. All emergency and outpatients poisoning were excluded from the study. Variables such as age, sex, marital status, length of hospital stay, year of hospital admission, season, mortality, and final diagnosis codes of all patients hospitalized for poisoning (46,202 cases) were extracted from the Hospital Information System (HIS) in excel format.

The 19th chapter of the ICD‐10 codes describes injury, poisoning, and certain other consequences of external causes. The 20th chapter refers to external causes of morbidity and mortality.^24^ The T36–T50 (drugs, medicaments, and biological substances) and T51–T65 (toxic effects of substances chiefly nonmedicinal) of ICD‐10 codes were used for the classification of patient diagnosis. We reported and discussed the trend of the five most frequent ICD‐10 categories codes of poisoning (34,810 cases) in detail such as T39, T40, T42, T43, and T50. The frequency of other subcategories was reported. In addition, the external causes of poisoning codes such as accidental poisoning by/or exposure to noxious substances (X40‐X49), intentional self‐harm (X60–X84), homicidal poisoning (X85‐X90), poisoning by undetermined intent (Y10‐Y19), and adverse drug effects (Y40‐Y59) were evaluated.

First, the data were cleaned. In the first step, the data was checked for possible duplicates based on the national identity, medical record number, age, and date of admission. Then the cases with incomplete or wrong‐recorded ICD‐10 codes of poisoning, wrong date of birth, and wrong date of discharge (compared to the date of admission) were omitted. Furthermore, any cases having wrong information due to incorrect coding were excluded.

Data were analyzed by SPSS version 16 (SPSS Inc.). For describing quantitative variables mean ± standard deviation was used while the categorical variables were presented as frequency and percentage. Furthermore, the rate of poisoning per 100,000 persons was calculated based on the total population of Mashhad each year. The population was extracted from the Population and Housing Censuses of Iran in 2016 conducted by the Statistical Center of Iran. The population for the remaining years was interpolated using the population projection formula.

## 3 RESULTS

Of the 46,202 patients, 11,392 were not included in the analysis because of having missing data on some variables or wrong coding during the registry. Of the 34,810 remaining patients, 50.7% of them were men. The majority of the patients were married (54.4%). The mean age of the patients was 29.64 ± 14.69 years, with a range of 3 days to 97 years, and men were somehow older than women (31.98 ± 15.66 years and 27.56 ± 13.19 years, respectively). In addition, the mean length of hospital stay for poisoned patients was 2.1 ± 4.7 days (men = 2.5 ± 5.5 and women = 1.7 ± 3.6). The total number of poison‐related deaths in the top five categories was 435 cases (320 males and 115 females). The distribution of poisonings in different seasons was similar (from 27.4% of cases in summer to 23.2% in winter) (Table 1).

**Table 1:** Characteristics of patients hospitalized for poisoning with five most common categories based on ICD‐10 codes admitted in Imam Reza hospital from 2012 to 2018

Variable		Men ratio (%)	Women ratio (%)	Total *N* (%)
Sex		17,652/34,810 (50.7)	17,158/34,810 (49.3)	(*n* = 34,810)
Marital status	Single	7955/17,652 (45.1)	5591/17,158 (32.6)	13,546/34,810 (38.9)
	Married	8764/17,652 (49.6)	10,190/17,158 (59.4)	18,954/34,810 (54.4)
	Divorced	413/17,652 (2.3)	784/17,158 (4.6)	1197/34,810 (3.4)
	Widow/widower	118/17,652 (0.7)	448/17,158 (2.6)	566/34,810 (1.6)
	Unknown	402/17,652 (2.3)	145/17,158 (0.8)	547/34,810 (1.6)
Year of hospital admission	2012	2408/17,652 (13.6)	2343/17,158 (13.7)	4751/34,810 (13.6)
	2013	3111/17,652 (17.6)	3318/17,158 (19.3)	6429/34,810 (18.5)
	2014	2795/17,652 (15.8)	2824/17,158 (16.5)	5619/34,810 (16.1)
	2015	3140/17,652 (17.8)	2797/17,158 (16.3)	5937/34,810 (17.1)
	2016	2651/17,652 (15.0)	2530/17,158 (14.7)	5181/34,810 (14.9)
	2017	2302/17,652 (13.0)	2165/17,158 (12.6)	4467/34,810 (12.8)
	2018	1245/17,652 (7.1)	1181/17,158 (6.9)	2426/34,810 (7.0)
Season	Spring	4538/17,652 (25.7)	4511/17,158 (26.3)	9049/34,810 (26.0)
	Summer	4874/17,652 (27.6)	4666/17,158 (27.2)	9540/34,810 (27.4)
	Fall	4173/17,652 (23.6)	3957/17,158 (23.1)	8130/34,810 (23.4)
	Winter	4067/17,652 (23.0)	4024/17,158 (23.5)	8091/34,810 (23.2)
Mortality		320/17,652 (1.8)	115/17,158 (0.7)	435/34,810 (1.2)

According to the codes blocks of the 19th chapter of ICD‐10, the five most frequent ICD‐10 categories and subcategories codes are presented in Table 2. The results of Table 2 show that the most common categories of poisoning were poisoning by antiepileptic, sedative‐hypnotic, antiparkinsonism drugs (T42), narcotics and hallucinogens drugs (T40), nonopioid analgesics, antipyretics and anti‐rheumatics drugs (T39), psychotropic drugs (T43) in descending order. Benzodiazepine poisoning (T42.4) had the highest frequency among other subcategories and it was more common among females than males (60.5% vs. 39.5%). Methadone poisoning (T40.3) was more common in men (67.2%). Opium poisoning (T40.0) has the highest mortality rate (5.4%) among other subcategories that is more common in males (72.0%). The mortality associated with narcotics had the highest frequency (2.7%).

**Table 2:** The frequency of the five most common categories and three most common subcategories codes of poisoning based on ICD‐10 in patients admitted to Imam Reza hospital from 2012 to 2018

ICD‐10 category and subcategory codes	Description	ratio (%)^a^	Sex ratio (%)^b^		Age mean ± SD	Mortality ratio (%)^b^
			Male	Female		
T39	Poisoning by nonopioid analgesics, antipyretics, and antirheumatics	10,412/34,810 (29.9)	5242/10,412 (50.3)	5170/10,412 (49.7)	24.94 ± 8.93	48/10,412 (0.5)
T39.0	Salicylates	290/34,810 (0.8)	76/290 (26.2)	214/290 (73.8)	27.38 ± 13.19	1/290 (0.3)
T39.1	4‐Aminophenol derivatives	3518/34,810 (10.1)	882/3518 (25.1)	2636/3518 (74.9)	25.05 ± 9.27	10/3518 (0.3)
T39.3	Other nonsteroidal anti‐inflammatory drugs [NSAID]	7665/34,810 (22.0)	4576/7665 (59.7)	3089/7665 (40.3)	24.75 ± 8.47	40/7665 (0.5)
T40	Poisoning by narcotics and psychodysleptics [hallucinogens]	10,599/34,810 (30.4)	7249/10,599 (68.4)	3350/10,599 (31.6)	35.06 ± 19.39	290/10,599 (2.7)
T40.0	Opium	3739/34,810 (10.7)	2692/3739 (72.0)	1047/3739 (28.0)	43.12 ± 22.26	202/3739 (5.4)
T40.3	Methadone	4600/34,810 (13.2)	3089/4600 (67.2)	1511/4600 (32.8)	33.23 ± 17.51	81/4600 (1.8)
T40.4	Other synthetic narcotics	1530/34,810 (4.4)	1091/1530 (71.3)	439/1530 (28.7)	28.42 ± 13.45	7/1530 (0.5)
T42	Poisoning by antiepileptic, sedative‐hypnotic and antiparkinsonism drugs	11,087/34,810 (31.9)	4285/11,087 (38.6)	6802/11,087 (61.4)	29.53 ± 12.36	84/11,087 (0.8)
T42.1	Iminostilbenes	446/34,810 (1.3)	150/446 (33.6)	296/446 (66.4)	26.40 ± 11.53	4/446 (0.9)
T42.4	Benzodiazepines	9250/34,810 (26.6)	3654/9250 (39.5)	5596/9250 (60.5)	29.99 ± 12.43	67/9250 (0.7)
T42.6	Other antiepileptic and sedative‐hypnotic drugs	1256/34,810 (3.6)	474/1256 (37.7)	782/1256 (62.3)	29.00 ± 11.28	10/1256 (0.8)
T43	Poisoning by psychotropic drugs, not elsewhere classified	5446/34,810 (15.6)	2273/5446 (41.7)	3173/5446 (58.3)	29.51 ± 11.85	59/5446 (1.1)
T43.0	Tricyclic and tetracyclic antidepressants	1502/34,810 (4.3)	402/1502 (26.8)	1100/1502 (73.2)	28.23 ± 11.26	10/1502 (0.7)
T43.2	Other and unspecified antidepressants	1500/34,810 (4.3)	390/1500 (26.0)	1110/1500 (74.0)	27.56 ± 10.58	3/1500 (0.2)
T43.6	Psychostimulants with abuse potential	1471/34,810 (4.2)	1084/1471 (73.7)	387/1471 (26.3)	32.51 ± 11.92	35/1471 (2.4)
T50	Poisoning by diuretics and other and unspecified drugs, medicaments, and biological substances	3836/34,810 (11.0)	1607/3836 (41.9)	2229/3836 (58.1)	28.92 ± 13.59	36/3836 (0.9)
T50.2	Carbonic‐anhydrase inhibitors, benzothiadiazides, and other diuretics	31/34,810 (0.1)	6/31 (19.4)	25/31 (80.6)	27.53 ± 16.32	0/31 (0.0)
T50.7	Analeptics and opioid receptor antagonists	131/34,810 (0.4)	111/131 (84.7)	20/131 (15.3)	35.00 ± 9.70	1/131 (0.8)
T50.9	Other and unspecified drugs, medicaments, and biological substances	3605/34,810 (10.4)	1468/3605 (40.7)	2137/3605 (59.3)	28.71 ± 13.58	35/3605 (1.0)

^a^ Out of all cases of top 5 (*n* = 34,810).

^b^ Out of all cases in each row.

Among the causes of poisoning, suicide (intentional self‐harm) (83.6%) was the most common cause, followed by undetermined intent (11.6%) and accidental consumption (3%), respectively.

**Figure 1:**
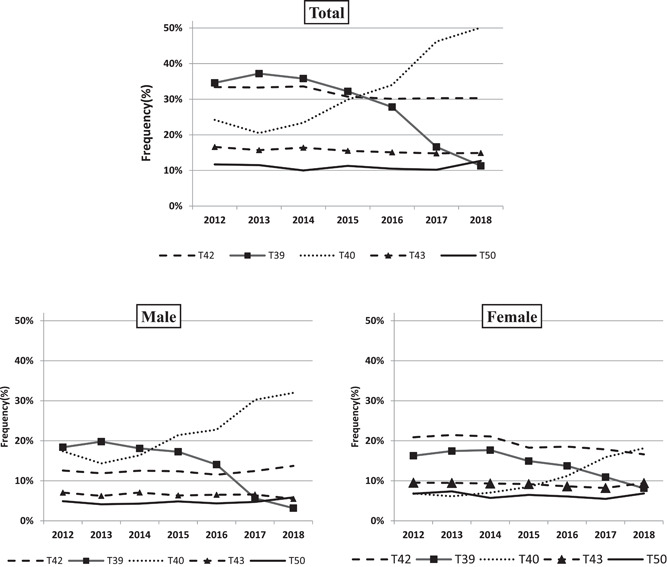
Trend of the five most common causes of poisoning (based on ICD‐10 codes) in patients admitted to Imam Reza hospital from 2012 to 2018 (Top: all patients, Bottom left: males, Bottom right: females)

The results in Figure 1 show that in general, poisoning with T40 drugs (such as Methadone and Opium) has increased and poisoning with T39 drugs (such as NSAIDs and 4‐Aminophenol derivatives) has decreased from 2012 to 2018 (top panel). This trend of poisoning was the same in men and women (bottom left and right panel).

**Figure 2:**
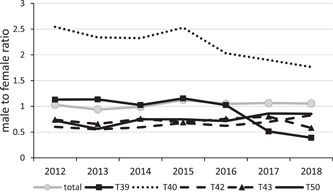
Trend in sex ratio (male/female) of five most causes of poisoning (based on ICD‐10 codes) in patients admitted in Imam Reza hospital from 2012 to 2018

As shown in Figure 2, poisoning with T40 drugs (such as Methadone) has increased dramatically in women compared to men from 2012 to 2018. In addition, poisoning with T39 drugs has significantly increased in women since 2016, while the overall sex ratio has been the same over the years. Poisoning with T42 drugs (such as Benzodiazepines) has increased by almost 30% in men, from 0.6 to 0.83 from 2012 to 2018.

**Figure 3:**
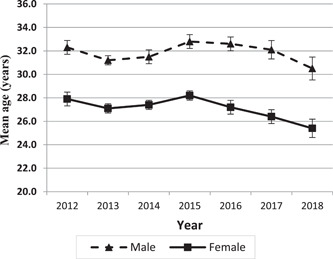
Mean age (years) along with 95% confidence interval of patients hospitalized for poisoning with the five most common substances based on ICD‐10 codes admitted to Imam Reza hospital from 2012 to 2018

Figure 3 shows that the average age of poisoned people of both sexes has decreased since 2015, and drug poisoning has occurred at younger ages.

**Figure 4:**
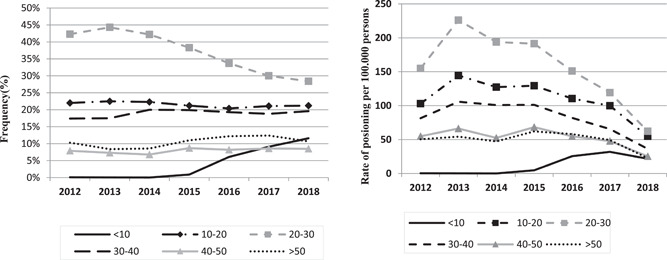
Frequency (left panel) and risk per 100,000 persons (right panel) of poisoning with five most common substances based on ICD‐10 codes of patients admitted to Imam Reza hospital from 2012 to 2018 in different age groups

Figure 4 shows that drug poisoning in these five categories has increased in children under the age of 10 since 2015. This upward trend can also be seen in the poisoning rate chart per 100,000 population. On the other hand, in all age groups except children under 10 years old, from 2012 to 2013, we have witnessed an increasing trend in the rate of poisoning based on the five common categories of T39, T40, T42, T43, and T50.

## 4 DISCUSSION

In this study, poisoning with drugs such as benzodiazepines and methadone was the most common cause of poisoning during 2012–2018. The mean age of poisoning in men and women has decreased, and these findings are consistent with the findings of other studies conducted in Iran and other countries.^17^, ^20^, ^21^, ^25^, ^26^, ^27^, ^28^ The prevalence of poisoning in young people can be due to socioeconomic conditions. This suggests that psychological interventions and the development of life skills can be very useful in reducing the prevalence of poisoning in young people.

Drug poisoning and abuse are considered among threatening dangers in many countries since they can have a negative effect on the health of people in society.^4^, ^29^ This study was conducted to investigate the epidemiology of poisoning trends in northeastern Iran. The trend of the five poisoning categories with the highest frequency in northeastern Iran was determined. Few studies have examined the trend of poisoning in this country. The pattern of poisoning and its cause vary from country to country, depending on the availability of toxic substances.^17^, ^30^

In this study, based on the top five categories, the highest frequency of poisoning was related to benzodiazepines (26.6%) and the highest incidence of poisoning was with this drug. Most of the people who were poisoned with benzodiazepines were women. Benzodiazepines are often used to treat anxiety, insomnia, and other conditions, and are more commonly prescribed to women than men. Emergency department visits and overdose deaths involving benzodiazepines have increased significantly among women in recent years.^31^ This finding can be attributed to the accessibility of pharmaceuticals products in Iran. Over‐the‐counter (OTC) access to benzodiazepines appears to play a major role in drug intoxication in the study population. Numerous studies have been performed in this regard in the past, and their findings are consistent with the findings of the present study.^20^, ^25^, ^27^, ^32^

The findings of the present study showed that poisoning by narcotics such as Opium (T40.0) and Methadone (T40.3) was common in men and some studies have been conducted in this regard that confirms this.^33^, ^34^ In Iran, morbidity, and mortality due to poisoning are more common in men due to easy access to medicine and drugs as well as their abuse.^4^ A common cause of mortality in men was poisoning by narcotics, such as Opium (T40.0). A study by Titidezh et al. determined that opium became the second most common poisoning agent and caused 88.6% of the deaths among patients referred to Baharloo Hospital in Tehran, Iran from 2011 to 2014.^4^ Lund et al. aimed at epidemiological investigation of poisoned individuals hospitalized in Oslo and found that opioids are the most common causes of death.^6^ In Iran, opium, and opium residue are the most common opioids, one of the reasons for which could be the place of Iran and Khorasan Razavi province on the transit path of drugs, including opium, which has facilitated access to this substance.

In this study, we found that intentional self‐harm (suicide) poisoning had the highest frequency among causes of poisonings. In addition, most of the poisoned patients were young people (almost 30 years old). It may also be due to easy access to drugs in Iran. Thus, policymakers should have more control over the sale of drugs without a physician's prescription. Numerous studies have been conducted that confirm the findings of the present study.^4^, ^15^, ^20^, ^21^, ^26^ There are numerous factors such as family conflicts, financial instabilities, unemployment, friendship losses, substance abuse, education failures, health‐related quality of life, legal problems, and mental health problems such as depression is associated with suicide from a medication overdose.^35^, ^36^ Cultural and economic problems can play an important role in suicide and poisoning.^4^

Mehrpour et al. aimed to assess the epidemiological and clinical profiles of acute poisoning in all adult patients admitted to the intensive care unit in eastern Iran (the city of Birjand) from 2010 to 2017 and found that pharmaceutical drugs (benzodiazepines and tricyclic antidepressants), opioids (methadone, opium, opium residue), and pesticides are the most common causes of poisoning. In addition, most of the poisonings were due to intentional self‐harm (suicide),^20^ which were consistent with our study findings.

Shokrzadeh et al. investigated the trend and characteristics of drug poisoning and compared them with other poisonings in Gorgan (north of Iran) from 2008 to 2015 and found that drug poisoning is the most common cause of poisoning. Benzodiazepines play an important role in drug poisoning. Also, they found that 77.1% of the cases were intentionally poisoned (with intent to commit suicide).^25^ The findings of our study were consistent with this study.

According to the findings of the present study, summer (27.7), spring (26.1), winter (23.2), and autumn (23.0) have the highest frequency of poisonings, respectively. These findings were consistent with the findings of the study by Mehrpour et al.^20^

One of the notable limitations in this study was that only patients for whom a hospitalized medical record was created and whose diagnosis was coded were included in the study, and all of the emergency and outpatients poisoning were not considered in this study, which could affect the data comprehensiveness. Another notable limitation is that the coding quality of poisonings was not examined in this study. However, since the coding of medical records in the hospital under study is done by health information management specialists, the researchers assumed the high accuracy of the assigned codes. In the review of the top five categories of poisoning, many other subcategories were observed, which could be considered as a limitation of ICD‐10 in the classification of drugs and chemicals, and this classification system needs to be updated. In addition, due to the incompleteness of some of the recorded data, we had to omit a large number of data from poisoned patients. As another limitation of this study, the launch of the child poisoning department in 2015 can be mentioned, and since then, child poisoning data are recorded.

## 5 CONCLUSION

In this study, it was found that poisoning due to some drugs such as benzodiazepine and methadone was the most common cause of poisoning. Most of the patients with poisoning are young and they had intentional self‐harm. It seems that reducing the availability of OTC drugs and decreasing the prescription of unnecessary pharmaceutical compounds, drug trafficking control, addiction rehabilitation therapies, and suicide prevention programs for younger ones can be helpful in poisoning reduction in this age group in this part of the country. Therefore, policymakers and healthcare managers can adopt the necessary programs and policies to prevent and control poisonings.

## AUTHOR CONTRIBUTIONS

*Conceptualization*: Alireza Banaye Yazdipour, Khalil Kimiafar, Masoumeh Sarbaz, and Bita Dadpour. *Formal Analysis*: Hamid Heydarian Miri, Saeedeh Hajebi Khaniki, and Mohammad Moshiri. *Funding Acquisition*: Khalil Kimiafar, Masoumeh Sarbaz, and Bita Dadpour. *Writing—review and editing*: Alireza Banaye Yazdipour, Khalil Kimiafar, Mohammad Moshiri, Masoumeh Sarbaz, Bita Dadpour, Hamid Heydarian Miri, and Saeedeh Hajebi Khaniki. *Writing*—*original draft*: Alireza Banaye Yazdipour. All authors have read and approved the final version of the manuscript. The corresponding author had full access to all of the data in this study and takes complete responsibility for the integrity of the data and the accuracy of the data analysis.

## CONFLICTS OF INTEREST

The authors declare no conflicts of interest.

## TRANSPARENCY STATEMENT

Alireza Banaye Yazdipour confirms that the manuscript is an honest, accurate, and transparent account of the study being reported; that no important aspects of the study have been omitted; and that any discrepancies from the study as planned (and, if relevant, registered) have been explained.

## ETHICS STATEMENT

The study was approved by the Ethical Committee of Mashhad University of Medical Sciences with Ethical number of IR. MUMS. REC.1395.160.

## Data Availability

The data that support the findings of this study are available from the corresponding author upon reasonable request. The data are not publicly available due to privacy or ethical restrictions.
